# Measuring the impact of zero-cases studies in evidence synthesis practice using the harms index and benefits index (Hi-Bi)

**DOI:** 10.1186/s12874-023-01884-x

**Published:** 2023-03-13

**Authors:** Chang Xu, Luis Furuya-Kanamori, Lifeng Lin, Liliane Zorzela, Tianqi Yu, Sunita Vohra

**Affiliations:** 1 Key Laboratory for Population Health Across-Life Cycle (Anhui Medical University), Ministry of Education, Anhui, China; 2 School of Public Health, Anhui Medical University, Anhui, China; 3 School of Public Health, Faculty of Medicine, University of Queensland, Brisbane, Australia; 4 Department of Epidemiology and Biostatistics, University of Arizona, Tucson, AZ USA; 5 Department of Pediatrics, Faculty of Medicine & Dentistry, University of Alberta, Edmonton, AB Canada; 6 Research Center of Epidemiology and Statistics (CRESS-U1153), INSERM, Université Paris Cité, Paris, France; 7 Department of Psychiatry, Faculty of Medicine & Dentistry, University of Alberta, Edmonton, AB Canada

**Keywords:** Zero-events studies; harms index, Benefits index, Robustness of the results, Evidence-synthesis practice

## Abstract

**Objectives:**

In evidence synthesis practice, dealing with studies with no cases in both arms has been a tough problem, for which there is no consensus in the research community. In this study, we propose a method to measure the potential impact of studies with no cases for meta-analysis results which we define as harms index (Hi) and benefits index (Bi) as an alternative solution for deciding how to deal with such studies.

**Methods:**

Hi and Bi are defined by the minimal number of cases added to the treatment arm (Hi) or control arm (Bi) of studies with no cases in a meta-analysis that lead to a change of the direction of the estimates or its statistical significance. Both exact and approximating methods are available to calculate Hi and Bi. We developed the “hibi” module in Stata so that researchers can easily implement the method. A real-world investigation of meta-analyses from Cochrane reviews was employed to evaluate the proposed method.

**Results:**

Based on Hi and Bi, our results suggested that 21.53% (Hi) to 26.55% (Bi) of Cochrane meta-analyses may be potentially impacted by studies with no cases, for which studies with no cases could not be excluded from the synthesis. The approximating method shows excellent specificity (100%) for both Hi and Bi, moderate sensitivity (68.25%) for Bi, and high sensitivity (80.61%) for Hi compared to the exact method.

**Conclusions:**

The proposed method is practical and useful for systematic reviewers to measure whether studies with no cases impact the results of meta-analyses and may act as an alternative solution for review authors to decide whether to include studies with no events for the synthesis or not.

**Supplementary Information:**

The online version contains supplementary material available at 10.1186/s12874-023-01884-x.

## Introduction

In evidence synthesis practice, dealing with studies with no cases in both arms (referred to as studies with no cases hereafter) has been a tough problem, for which there is no consensus in the research community. The current practice is to discard such studies from the synthesis when measures of relative effects (i.e., risk ratio, odds ratio) are utilized [1]. This is because some researchers believe that studies with no cases add nothing to the pooled effects as their conditional likelihoods are constants (i.e., no contribution to the parameter estimation) [2]. Another reason is that researchers compared the performance of discarding such studies versus including such studies through classical methods (e.g., continuity correction) and found that discarding them seemed to yield better statistical properties [3, 4].

However, this approach is not always true due to several reasons. First, studies with no cases are not necessarily “non-informative,” depending on the methods used for meta-analysis. For example, some methods based on the one-stage framework are suitable to include such studies and provide evidence that those studies contain information for statistical inference [5–9]. The synthesis methods and simulation mechanisms also affect the statistical properties for discarding such studies versus including them [10]. For example, within the one-stage framework, discarding and including such studies have comparable performance [11]. Second, when using measures of absolute effect, say, the difference of likelihood, as effect estimates, studies with no cases matter for statistical inference. Third, from the clinical point of view, for studies with balanced sample sizes in two arms, no cases in both arms indicate no difference in risks (or benefits), which is certainly an important source of evidence for clinical practice [8].

Despite the many recent efforts devoted to this research direction, dealing with studies with no cases in meta-analysis is still a difficult task from both the methodological and applied perspectives. In our recent publication [12], we proposed a framework to classify meta-analysis with zero-cases studies into six subtypes. Although this framework summarizes suitable methods for dealing with zero cases studies for each subtype, in some situations, the available statistical methods are limited, and many of the methods are sophisticated and complicated. We have argued that there are no methods readily available for users that can be broadly applied to all subtypes. For most researchers, it is challenging to fully understand and accurately implement these sophisticated methods. These produce a dilemma for handling studies with no cases: discarding them would lead to research waste and may mislead decision-making, while including them is difficult to implement.

In this article, we propose a “compromise” solution, namely the harms index (Hi) and benefits index (Bi), by measuring the potential impact of studies with no cases on the pooled effect. Our key proposal is that when there is no (or little) impact of studies with no cases on the pooled effect, researchers can discard these studies in the formal synthesis but need to mention their existence when reporting the systematic review and meta-analysis. On the other hand, when these studies impact the pooled effect, researchers need to routinely include them in the formal synthesis.

## Methods

### Applicability

**Fig. 1:**
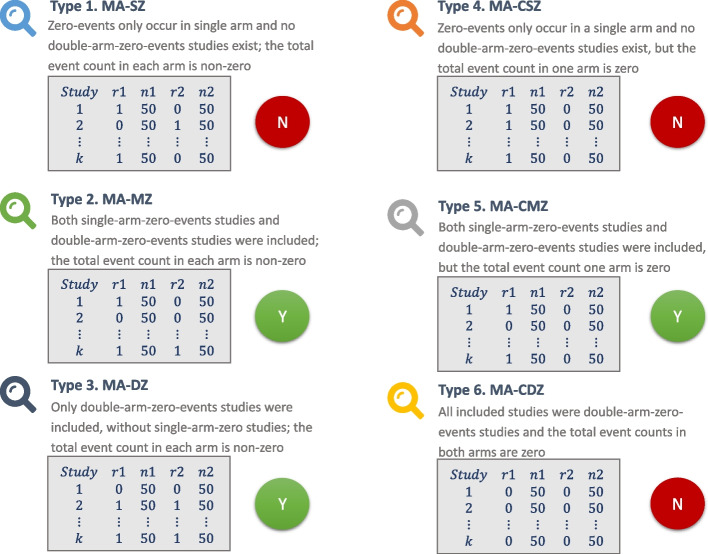
Applicability of Harms index (Hi) and Benefits index Bi

The idea of this paper is driven by Atal et al.’s discussion on the fragility index for meta-analysis [13], which assesses the robustness of statistical significance of the pooled effect. Unlike the fragility index, our Hi and Bi are aimed at measuring the potential impact of studies with no cases on the final effect in a meta-analysis. They are applicable to a meta-analysis containing at least one study with no cases, as well as at least one study with cases in either or both arms. This means that studies with zero cases in only one of the arms (single-arm-zero-cases studies) are not considered a “study with no cases”; instead, only studies with zero cases in both arms (double-arm-zero-cases studies) are considered a “study with no cases”. Based on our framework for meta-analysis with zero cases studies [12], three subtypes, including the meta-analysis with double-zero-cases studies (MA-DZ), meta-analysis with mixture-zero-cases studies (MA-MZ), and meta-analysis with completely mixture-zero-cases studies (MA-CMZ), are suitable for the Hi and Bi method. For the other three subtypes, i.e., the meta-analysis with single-zero-cases-studies (MA-SZ), meta-analysis with completely single-zero-cases-studies (MA-CSZ), and meta-analysis with completely double-zero-cases studies (MA-CDZ), there is no need to measure the impact of excluding studies with no cases. For MA-SZ and MA-CSZ, only single-arm-zero-cases studies are involved, and Peto’s OR method and MH method work well for the synthesis. For MA-CDZ, the effect is evident that there is no difference in the risks of the two arms. Figure 1 presents the applicability of the Hi and Bi.

### Definition of harms index (Hi) and benefits index (Bi)

We defined the Hi as the minimum number of cases added in the treatment arm for studies with no cases that can change the direction of the effects or the significance of the meta-analysis. Of note, in this process, the sample size in each arm of each study remains unchanged, so the number of non-cases is accordingly decreased. The addition of cases in the treatment arm pushes the effect to the “harm” side; hence, we call it the harms index. We defined the Bi as the minimum number of cases added in the control arm for studies with no cases that can change the direction of the effects or the significance of the meta-analysis. We further defined the direction of the effects as the pooled estimates (e.g., OR, RR) change from > 1 to < 1 or vice versa; the direction of the significance as the *P* value change from statistically significant to non-significant or vice versa. Contrary to the Hi, the addition of cases in the control arm will push the effect to the “benefit” side, so we call it the benefits index. There is a distinction between the “cases” and the “events”; a single individual may have more than 1 event during the intervention (e.g., 10 patients may have 20 events), while the same individual can only be at most 1 case. This is the reason that we use the term “cases” here instead of “events” throughout the manuscript.

The Hi and Bi are defined by two elements, i.e., the number of cases added in the studies with no cases and the number of studies with no cases. To be more accurate, the Hi and Bi are the minimum number of cases multiplied by the number of studies added in the treatment or control arms. For example, for a meta-analysis, if adding 1 case, at minimum, to the treatment arm of each of the two studies with no cases change the effect direction (or significance), then the Hi is 1*1 + 1*1 = 2. This derivation can be generalized as the following formulas:

$$Hi=\sum_{j=1}^kt\_{cases}_j;Bi=\sum_{j=1}^kc\_{cases}_j.$$

Here, $k$ is the minimal number of studies with no cases required to drive a change of the direction of the effects or the significance, $t\_{cases}_{j}$ (*j* = *1, …, k*) indicates the number of cases added in the treatment arm in the $j$ ^*th*^ study with no cases. Similarly, $c\_{cases}_{j}$ indicates the number of cases added in the control arm in the $j$ ^*th*^ study with no cases. Considering that larger studies with no cases tend to have a larger impact on the pooled effect, the adding-cases procedure is first implemented in the largest studies with no cases, and then the second largest, until the smallest one.

**Fig. 2:**
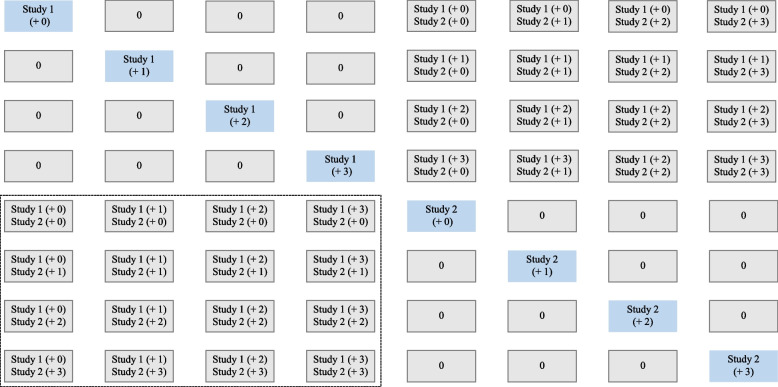
Combinations when there are two studies with no cases in a meta-analysis

Provided we have $n (n \ge k)$ studies with no cases, and $m (m \ge t\_{cases}_{j}; m \ge c\_{cases}_{j})$ cases could be added at most. The detailed process of calculating the Hi is as follows:

1. Conduct the meta-analysis by excluding studies with no cases, and obtain the pooled effect ${\widehat{\theta }}_{0}$ and the p-value ${\widehat{p}}_{0}$ (reference combination).
2. Rank the studies with no cases by the sample size, subsequently add 1 to m cases in the treatment arm of the largest study with no cases, and re-do the meta-analysis by including this study.
3. Reset the cases of the largest studies with no cases to zero, and subsequently add 1 to m cases in the treatment arm of the second largest study with no cases, and re-do the meta-analysis by including this study. Repeat the process until the smallest study with no cases.
4. Add 1 to m cases in the treatment arm for two of the studies (if $n\ge 2$) with no cases, while setting the arm of the third study (if existed) with no cases as zero; exhaust all possible such two-two combinations. In this way, exhaust all possible combinations from two-two to n–n and re-do meta-analysis for all of the combinations. For the above four processes, there are $({m+1)}^{n}$ combinations, including the one reference combination (see Fig. 2 and Figure S1).
5. Compare the direction of the pooled effects and the p-values of all non-reference combinations to the reference combination (${\widehat{\theta }}_{0}$ and ${\widehat{p}}_{0}$).
6. Rank the combinations, and identify the first combination with the pooled effect direction or significance changed, where the number of cases multiplied by the number of studies is the value of Hi.

The calculation of Bi is similar to the above process for the Hi; the only difference is that for the Bi, the cases are added to the control arm. According to the definition, larger Hi and Bi imply that more additional cases are needed to lead to a change, which further indicates that the meta-analysis is more stable to studies with no cases.

When no combination changes the direction of the effects as well as the significance, the Hi/Bi is defined to be 0. We should be clearer with the situations where Hi = 0 while Bi > 0 and Hi > 0 while Bi = 0. The former means adding cases in the treatment arm does not impact the effect, while adding cases in the control arm may push the effects to be more beneficial. The latter means adding cases in the treatment arm may push the effect to be more harmful, while adding cases in the control arm does not impact the effect. It should be noted that only if Hi = Bi = 0, we can conclude that studies with no cases do not impact the pooled effect direction and significance of the meta-analysis.

### The maximum cases added to studies with no cases

The natural maximum cases (m) that could be added to studies with no cases is the sample size of the arm. However, this natural range is unrealistic since the true case rate would be very low (the observed rate is 0 for these studies), so it is not reasonable in terms of probability to consider adding too many cases to studies with no cases. Moreover, a larger m would require a large amount of computational effort. For example, suppose $m=3$ (means we can add 1, 2, or 3 cases to the studies with no events); and there are three studies with no cases, then there would be (3 + 1)^3 = 64 combinations, corresponding to 64 meta-analyses involved. If $\mathrm{m}$ is increased to $5$ and to 10, there would be (5 + 1)^3 = 216 and (10 + 1)^3 = 1331 combinations, involving 216 and 1331 meta-analyses, respectively. It should be noted that the amount of computational effort also increased with the number of studies with no cases.

The rule of three ($\mathrm{m}=3$) provides us with a reasonable foundation to define the maximum cases. According to the rule of three, the upper 95% confidence interval of the rates of a zero-case sample is 3/n [14, 15]. This means for 100 independent studies with no cases, 95 of them with the expected maximum cases in the arms would not exceed 3. Therefore, we propose to limit the maximum number of cases added to each arm to 3 for studies with no cases. Formally, for the calculation of Hi and Bi, the following condition is used:

$$Hi=\{\sum_{j=1}^kt\_{cases}_j\vert{t\_cases}_j\leq3\};Bi=\{\sum_{j=1}^kc\_{cases}_j\vert c\_{cases}_j\leq3\}.$$

Based on this rule, we can set a reasonable cutoff point of Hi and Bi to reflect whether the results of a meta-analysis are likely to be influenced by studies with no cases. In terms of probability, adding 3 or more cases in a study has less than 5% possibility. This means if adding 3 cases still does not lead to a change of the direction of the effect or the significance, there would be a very low possibility of adding more cases to studies with no cases, so the meta-analysis will not be susceptible to studies with no cases. A stricter cutoff point could also be considered as 5 [-ln (0.007)], which corresponds to a 99.3% confidence interval. Therefore, for Hi, based on the cutoff point, the impact of studies with no cases could be divided into three categories: 1) having no impact on the results of meta-analysis (Hi = 0); 2) almost having no impact on the results of meta-analysis (Hi > 3); 3) having a potential impact on the results (0 < Hi $\le$ 3). For Bi, the cutoff points and recommendations are similar to the above for Hi.

### Practical rules for evidence synthesis

In practice for evidence synthesis, according to the three categories of the impact, when there is no or almost no impact, researchers can discard studies with no events in the formal synthesis in order to simplify the synthesis process but need to mention their existence when reporting the results.

On the other hand, when these studies have a potential impact on the pooled effect, researchers need to include them in the formal synthesis. The methods for synthesizing studies with no events can be found in our previous paper [12]. In addition, for transparency, researchers should report the type of data structure based on the aforementioned framework [12], the number of studies with no events, the Hi-Bi value, and the methods used for dealing with studies with no events in both the abstract and full-text.

### Implementation of the Hi-Bi method

We developed the “*hibi*” module in Stata so that researchers can easily implement the Hi and Bi methods [16]. This module provides two types of meta-analytic methods, i.e., the one-stage method based on the beta-binomial model [17] and the two-stage method based on Peto’s OR [18], for calculating the Hi and Bi. The default effect estimate is Peto’s OR, because the one-stage method frequently occurs the problem of non-convergence and generally takes more time for the computation. We also provide the options for RR; Peto’s method cannot be used for RR, so the two-stage MH method is used instead. In addition, this module also provides the Hi plot and Bi plot to visualize the iteration process and the results.

In the “hibi” module, considering the potentially huge amounts of computations under the situation of many studies with no cases (e.g., *n* = 10 needs 4^10 = 1,048,576 times of meta-analyses for calculating the Hi and Bi respectively), we provide an approximating method for the calculation of the Hi and Bi. This method only considers the study with the largest weighted effect (default by Peto’s OR after adding 1 case to the treatment arm) among all studies with no cases, which would have the largest possibility to lead to the change of the direction of the effect or significance of the meta-analysis. The maximum number of cases added to the study changes to 3*n, so this process only involves (3*n + 1) times of meta-analyses. For example, if there are 10 studies with no cases, then in the approximating method, the maximum number of cases added to the “largest” study with no cases is 3*10 = 30, and only 31 times of meta-analyses are needed for calculating the Hi and Bi, respectively. It should be noted that, when the sample size of the arm of the “largest” study is less than the maximum number of cases, this maximum number is automatically replaced by the sample size of the arm of the study. We suggest using the approximating method only in the presence of $\ge$ 7 studies with no cases to save computational power.

### Examples

**Fig. 3:**
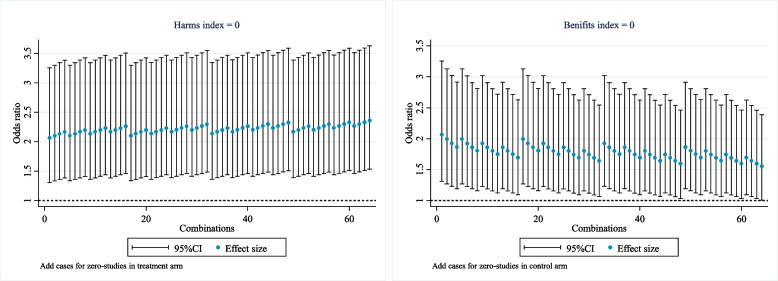
Example of Hi and Bi for the meta-analysis of serious adverse cases of surgery with a tourniquet vs surgery without a tourniquet

In a recent Cochrane systematic review, Ahmed et al. investigated the use of tourniquet in knee replacement surgery [19]. One of the outcomes is serious adverse cases of surgery with a tourniquet vs. surgery without a tourniquet. The outcome involves 21 studies, where 9 are single-arm-zero-cases studies, 3 are double-arm-zero-cases studies, and no arms have total counts of zero (Table S1). According to our framework [12], this meta-analysis belongs to the MA-CMZ, and thus is suitable to investigate the potential impact of studies with no cases on the results. In this example, the number of studies with no cases is not large, so the exact Hi and Bi method is utilized. Our results show that both Hi and Bi by the two-stage Peto’s OR are 0 (Fig. 3), which indicates that even adding 3 events to all three studies still does not lead to a change of the direction of the effect or significance, and therefore the result of the meta-analysis is reasonably robust to studies with no cases.

### Real-world investigation

We collected data from the Cochrane Database of Systematic Reviews from 2003 to 2018 for those binary meta-analyses that contained at least 1 study with no cases. A detailed description of the data collection process has been published previously [20–22]. In brief, a program based on R software was developed to download the rm5 files for each Cochrane review automatically, containing the metadata for each review. The rm5 files were further transferred into csv files that formed the current dataset (a flow diagram is presented in Figure S2). From the 6781 Cochrane reviews, we collected a total of 61,090 meta-analyses with binary outcomes, of which 658 contained at least 1 study with no cases. We further excluded 108 MA-CDZ and 108 MA-CSZ, leaving 442 meta-analyses eligible for the analysis in this article [11]. The number of studies with no cases ranged from 1 to 33, with a median number of 2 (first to third quartile: 1 to 3), while 10% of the meta-analyses had more than 8 studies with no cases (Table S2). Considering that meta-analyses with 6 or more studies with no cases would need a large amount of computational effort, we utilized those meta-analyses having 6 or fewer studies with no cases (N = 386) to compare the Hi and Bi by the exact method with those by the approximating method. Both sets of analyses were based on the two-stage Peto’s OR.

**Fig. 4:**
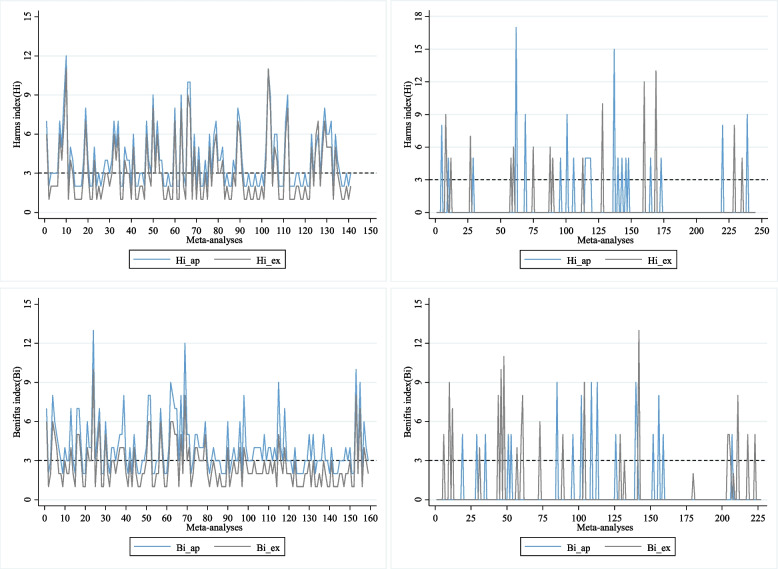
The real-world investigation of the Hi and Bi based on Cochrane reviews. 1) The left top panel presents Hi by the approximating (Hi_ap) and exact method (Hi_ex) when Hi > 0 by both methods; 2) The right top panel presents Hi by the approximating (Hi_ap) and exact method (Hi_ex) when Hi = 0 by either of the methods; 3) The left bottom presents Bi of the approximating (Bi_ap) and exact method (Bi_ex) when Bi > 0 by both methods; 4) The right top presents Bi of the approximating (Bi_ap) and exact method (Bi_ex) when Bi = 0 by either of the methods

Figure 4 shows the exact and approximate Hi and Bi. According to the results by the exact method, there were about 21.53% (Hi) to 26.55% (Bi) of the meta-analyses were potentially impacted by studies with no cases, and thus for these meta-analyses such studies could not be excluded. For the remaining 73.45% of the meta-analyses that were insusceptible to studies with no cases, such studies could not be synthesized to the results while a reporting of the reason should be mentioned. The approximating method produced higher Hi and Bi than the exact method. More specifically, when Hi > 0 by both the approximating and exact method, in 95.04% of the situations, the approximating method needed one more case than the exact method for producing the Hi; When Bi > 0 by both the approximating and method, in 72.96% of the situations, the approximating method needed one more case than the exact method for producing the Bi. This is not the case when Hi = 0 in either the approximating method or the exact method – in 84.9% of the situations, they had the same Hi; and when Bi = 0 in either the approximating method or the exact method, in 81.94% of the situations they had the same Bi.

**Fig. 5:**
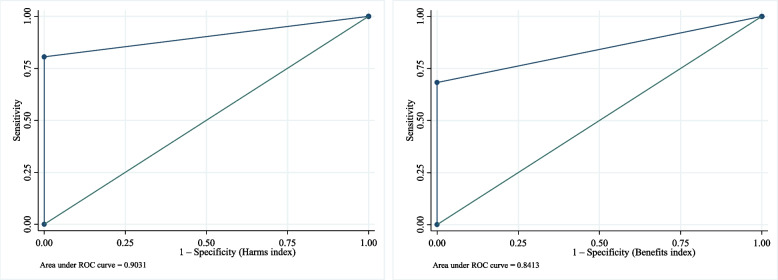
ROC for approximating method

Figure 5 presents the diagnostic test for the approximating method. In general, it performs well compared to the exact method (gold standard). For Hi, the sensitivity of the approximating method was 80.61%, and the specificity was 100%, with the ROC AUC of 0.90; this means for Hi, the approximating method detected 80.61% of the meta-analyses for which the results may be impacted by studies with no cases, while it detected 100% of the meta-analyses for which the results are not impacted by studies with no cases. For Bi, the sensitivity of the approximating method was 68.25%, and the specificity was 100%, with the ROC AUC of 0.84. Again, this means for Bi, the approximating method detected 68.25% of the meta-analyses for which the results are impacted by studies with no cases, while it detected 100% of the meta-analyses for which the results are not impacted by studies with no cases.

## Discussion

In this study, we proposed the harms index and benefits index to measure the potential impact of studies with no cases on the results of meta-analyses. To the best of our knowledge, this is the first attempt to measure the robustness of meta-analysis in terms of studies with no cases. Considering the difficulties in dealing with studies with no cases in meta-analyses, this method provides a reasonable solution for researchers trying to decide whether studies with no cases can be discarded from their evidence synthesis. In addition, we provided an approximating method for Hi and Bi when the number of studies with no cases is large. Based on the real-world dataset, our method suggested that the approximating method worked well, especially for Hi. In addition, for meta-analysis with at most 6 studies with no cases, about 21.53% (Hi) to 26.55% (Bi) of the meta-analyses were potentially impacted by studies with no cases, and thus for these meta-analyses, studies with no cases should not be excluded from the synthesis.

The harms index and benefits index may differ when different meta-analytic methods are employed [23–25]. For example, under a fixed-effect model (e.g., Peto’s OR), we may observe different Hi and Bi from in a random-effects model. This is because – as is widely recognized, the random-effects model tends to be more conservative than the fixed-effect model in the presence of heterogeneity. In addition, the way of dealing with single-arm zero-cases also has some impact on the calculation of Hi and Bi. For example, the continuity correction introduces additional sample size to the meta-analysis and thus may lead to the shrinkage of variance. Therefore, the statistical properties of these meta-analytic methods affect the properties of Hi and Bi. This is the reason that in the “hibi” module, we set Peto’s OR as the default method – many simulation studies have verified that Peto’s OR performs better than other two-stage methods in meta-analyses with single-arm-zero-cases studies [26–28].

It should be highlighted that the Hi and Bi only reflect whether studies with no cases would impact the effects of the meta-analysis. Even though the results of Hi and Bi suggest that studies with no cases could be excluded from the synthesis, it does not mean that studies with no cases are non-informative. Instead, it means studies with no cases are not necessarily non-informative, while the information of these studies would not alter the conclusions of the meta-analysis – in order to facilitate the evidence synthesis process, one can choose not to synthesize them in the meta-analysis in a “rapid synthesis” paradigm [29–31]. As mentioned earlier, when evidence suggests studies with no cases do not impact the results of the meta-analysis, researchers still need to mention them when reporting systematic reviews and meta-analyses as a reminder that such studies should not be ignored [32].

Some limitations of the Hi and Bi method should be noted. Perhaps the major limitation of Hi and Bi is that the computational process is often time-consuming and computationally expensive. In our experience, when a meta-analysis contains 6 or more studies with no cases, the process takes hours to obtain the exact Hi and Bi values. Five studies with no cases involve 4^6 = 4096 combinations, and all of these combinations are required for meta-analysis. This problem is more obvious when one-stage methods are employed due to the large amounts of iterations. In this situation, researchers can choose the approximating method to obtain the Hi and Bi. Another limitation is that we did not account for the impact of sample size ratio (i.e., treatment arm vs. control arm) for the estimation of Hi and Bi into consideration. For studies with no cases in a meta-analysis, when the sample size ratios are balanced (ratio $\approx 1$), adding the same number of cases for each study with no cases generally has the same weighted effect. However, when the ratios are seriously unbalanced, the weighted effects of each study with no cases differ a lot and can have different impacts on the results, particularly when the effect is small (e.g., OR < 1.5 or OR > 0.8). However, as long as there is an effect as moderate or large, this is not likely to substantially impact the calculation of the Hi and Bi. Further efforts are worthwhile to address these potential limitations.

In conclusion, the proposed method is practical and useful for systematic reviewers to measure whether studies with no cases impact the results. It could be treated as an alternative solution to dealing with meta-analysis with studies with no cases, rather than excluding such studies directly. We suggest further systematic review authors report the Hi and Bi values in their meta-analysis when studies with no cases are involved.

## Supplementary Information

**Additional file1:** **Figure S1.** All combinations (study 1, 2, 3… are studies with no cases). **Figure S2.** Diagram for real-world dataset. (From Xu C, Li L, Lin L, Chu H, Thabane L, Zou K, Sun X. Exclusion of studies with no events in both arms in meta-analysis impacted the conclusions. J Clin Epidemiol. 2020; 123:91-99.). **Table S1.** Serious adverse events for surgery with tourniquet vs. without tourniquet. **Table S2.** Baseline characteristics of the real-world dataset (442 meta-analyses with 3652 trials).

## Data Availability

All data generated or analysed during this study are included in this published article and its supplementary information files.
