# Supplementary material for: Measuring the impact of zero-cases studies in evidence synthesis practice using the harms index and benefits index (Hi-Bi)
Source: BMC Med Res Methodol. 2023 Mar 13;23:61. doi: 10.1186/s12874-023-01884-x (PMC10010026; doi:10.1186/s12874-023-01884-x)
Supplement: Supplementary file 1 — Additional file1: Figure S1. All combinations (study 1, 2, 3… are studies with no cases). Figure S2. Diagram for real-world dataset. (From Xu C, Li L, Lin L, Chu H, Thabane L, Zou K, Sun X. Exclusion of studies with no events in both arms in meta-analysis impacted the conclusions. J Clin Epidemiol. 2020; 123:91-99.). Table S1. Serious adverse events for surgery with tourniquet vs. without tourniquet. Table S2. Baseline characteristics of the real-world dataset (442 meta-analyses with 3652 trials). [file 12874_2023_1884_MOESM1_ESM.docx]

**Figure S1. All combinations (study 1, 2, 3… are studies with no cases)**

**
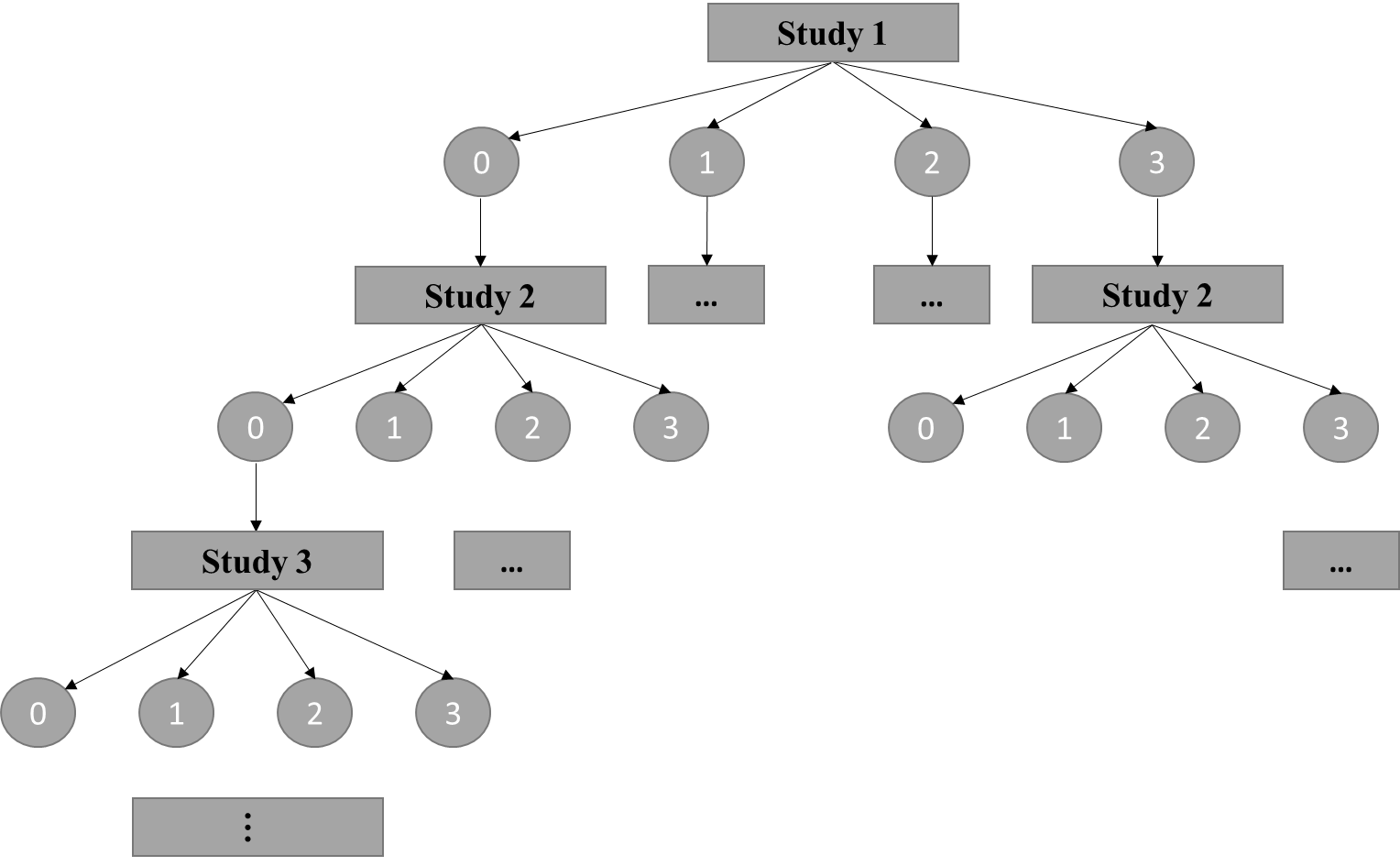
**

**Table S1.** Serious adverse events for surgery with tourniquet vs. without tourniquet.

| **Study** | **With tourniquet** | | **Without tourniquet** | |
| --- | --- | --- | --- | --- |
|  | **Events** | **Total** | **Events** | **Total** |
| Abdel-Salem 1995 | 5 | 40 | 0 | 40 |
| Alexandersson 2019 | 2 | 38 | 0 | 43 |
| Ejaz 2015 b | 2 | 33 | 1 | 31 |
| Goel 2019 | 3 | 100 | 3 | 99 |
| Huang 2017 | 1 | 50 | 0 | 50 |
| Jawhar 2020 | 3 | 50 | 2 | 49 |
| Kato 2002 | 1 | 22 | 0 | 24 |
| Li 2008 | 2 | 30 | 0 | 30 |
| Liu 2017 | 1 | 26 | 0 | 26 |
| Liu 2017 b | 4 | 56 | 4 | 56 |
| Matziolis 2004 | 1 | 10 | 0 | 10 |
| Molt 2014 | 0 | 30 | 2 | 30 |
| Mori 2016 | 0 | 51 | 0 | 52 |
| Tetro 2001 | 4 | 33 | 1 | 30 |
| Vandenbussche 2001 | 1 | 40 | 2 | 40 |
| Wakankar 1999 | 7 | 37 | 6 | 40 |
| Wauke 2002 | 2 | 19 | 0 | 18 |
| Wu 2018 | 0 | 50 | 0 | 50 |
| Zhang 2010 | 0 | 30 | 0 | 30 |
| Zhang 2016 | 9 | 84 | 2 | 82 |
| Zhou 2011 | 5 | 72 | 3 | 68 |

**Figure S2. Diagram for real-world dataset.**


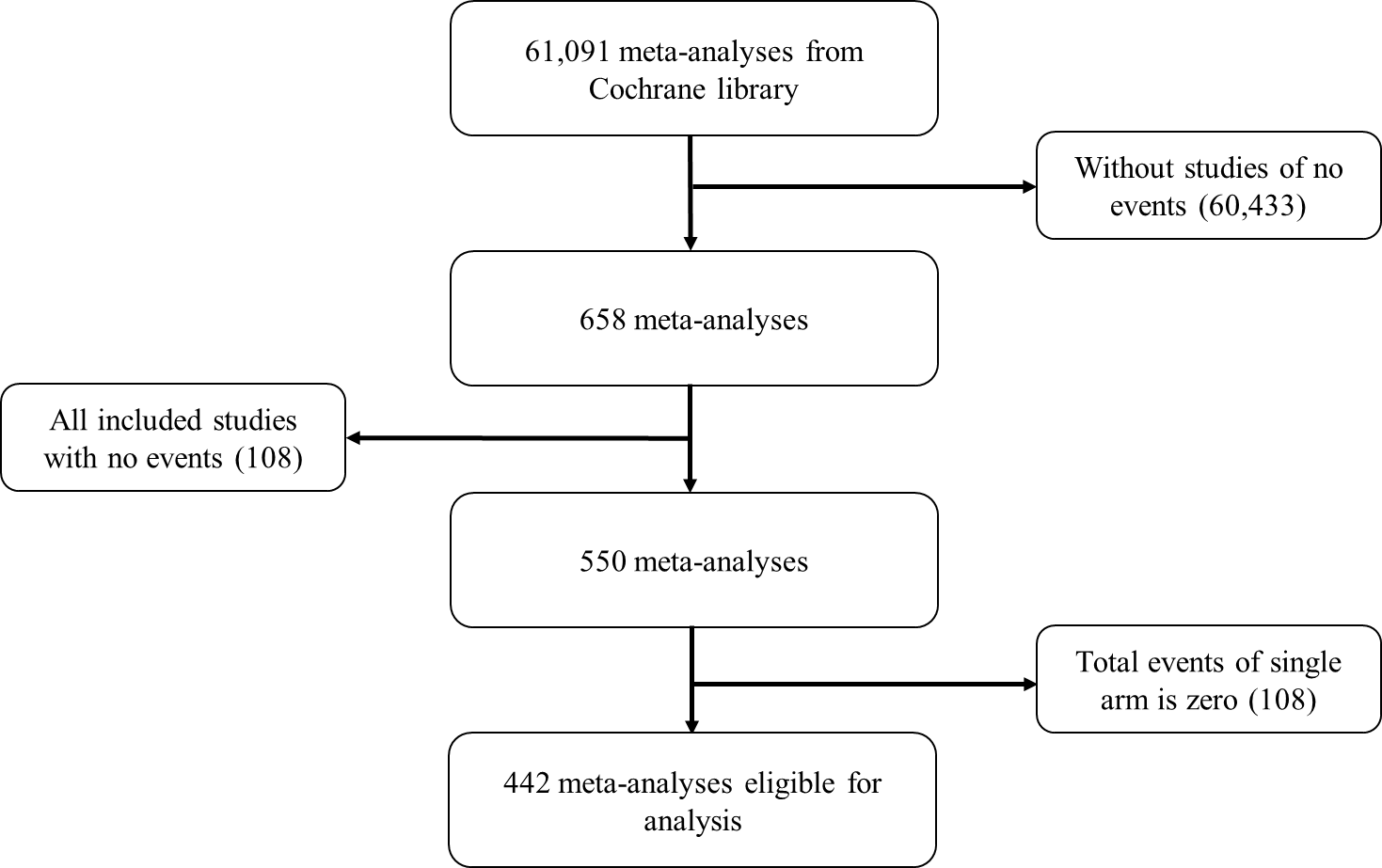


*(From Xu C, Li L, Lin L, Chu H, Thabane L, Zou K, Sun X. Exclusion of studies with no events in both arms in meta-analysis impacted the conclusions. J Clin Epidemiol. 2020; 123:91-99.)*

**Table S2.** Baseline characteristics of the real-world dataset (442 meta-analyses with 3652 trials).

| **Baseline characteristics** | **Meta-analyses with studies of no events** |
| --- | --- |
| **Number of studies within a meta-analysis** (ranges: 2 to 78) | 6 (4 to 10) |
| total range | 2 to 78 |
| <=6 | 244 (55.33%) |
| >6 | 197 (44.67%) |
| **Number of studies without events within a meta-analysis** (ranges: 1 to 33) | 2 (1 to 3) |
| <=2 | 284 (64.40%) |
| >2 | 157 (35.60%) |
| *Proportion for studies without events to the all (ranges: 3.57% to 92.31%)* | 42.86% (25.00% to 57.14%) |
| **Sample size in each meta-analysis** (ranges: 40 to 18034) | 516 (276 to 1206) |
| <=516 | 222 (50.23%) |
| >516 | 220 (49.77%) |
| **Sample size of studies without events in each study** (ranges: 2 to 1397) | 38 (22 to 71) |
| <=38 | 762 (20.83%) |
| >38 | 2890 (79.17%) |
| *Proportion for studies without events to the all within a meta-analysis (ranges: 1.15% to 97.50%)* | 21.78% (10.10% to 39.45%) |

*(From Xu C, Li L, Lin L, Chu H, Thabane L, Zou K, Sun X. Exclusion of studies with no events in both arms in meta-analysis impacted the conclusions. J Clin Epidemiol. 2020; 123:91-99.)*

**Program to estimate Hi-Bi value in multiple meta-analyses (based on the real-world investigation dataset)**

***Maid indicate the id of each meta-analysis; r1, c1, n1, and r2 c2, n2, indicate the cases, non-cases, total in each arm.

*egen maid1=group(maid)*

*drop maid*

*rename maid1 maid*

*gen c1 = n1-r1*

*gen c2= n2-r2*

*gen Hi =.*

*gen Bi =.*

*forvalues i = 1(1)442 {*

*hibi r1 c1 r2 c2 if maid ==`i', or nograph*

*replace Hi = _Hi if maid ==`i'*

*replace Bi = _Bi if maid ==`i'*

*drop _Hi _Bi*

*}*

***
